# Abdomen—Wheezing

**Published:** 1884

**Authors:** 


### SUPPLEMENT

[*Containing symptoms and remedies omitted from the repertory proper, also those not repeated under proper headings, and those less frequently met with.*]

---

**ABDOMEN, pain** in, dry, fatiguing cough from titillating in larynx; worse after midnight, in morning, and after eating, causing pain in stomach and soreness in abdominal walls: *Nux v.*[7]

— **pain** in umbilical region, very intense when coughing or stooping: Sepia.[6]

**AIR, open.** See also Walking in Open Air, p. 137.

**AIR PASSAGES**, cough from dull cutting, which becomes a stitch, in the air passages, from below upward, with mucus in chest: Arg.[7]

— **stuffed** up, influenza, air passages feel; dry, hard cough, stiffness in back and limbs: Rhus.[7]

**ANGER**, easily angered, and from it cough and stitches in chest: Arg. n.[7]

**ANUS**, stitches in rectum when coughing: Nitr. ac.[6]

**APPETITE**, capricious in a case of chronic cough and dyspnœa: Kali b.[20]

**ARMS**, dry cough with pain in arm and shoulders: Digit.[6]

— during cough, sudden pain in lower right arm, striking from above downward: Puls.[6]

— stitches in chest with cough and heat on the lower chest and upper arm: Calc. ph.[7]

**BARKING** cough: Ant. t.,[24] Caps.,[6] Lyssin. (Hg.), Merc. ac.[1]

— cough, dry, from tickling in larynx and pit of stomach; worse at night, also in day when lying down: Nitr. ac.[7]

— cough waking suddenly, 11 P. M.; face fiery red, crying with cough: Bell.[7]

**BATHING**, excites cough: Nux m.[7] See Washing.

**BED**, changing position in, amel. cough: Ign.[2]

— **lying** in, excites cough: Agnus, Crot. t.

— — — tickling in throat and cough morning, preventing him from lying in bed: Coc. c.[1]

— after **rising** from bed in morning, agg. of cough: Plumb.[7]

— on **rising** from bed, cough [illegible]rs.

— **sitting** up in. See Sit Up, p. 96.

— — cough when lying in bed, must sit up or sleep in chair from sense of suffocation: Crot. t. (J. C. Robert.)

— **warm** in, cough amel. on becoming: Kali b.[7]

**BELCHING.** Compare with Eructation, p. 39.

— after the cough: Sul. ac.[7]

— before and after coughing: Sang.[7]

— causes a scratching in throat, irritable larynx, then is followed by a cough: Staph.[7]

**BELCHING** or straining to vomit during an attack of cough: Arg. n.[7]

— cough with belching, gagging or vomiting: Cimex.[7]

**BODY**, cough in sudden paroxysms, convulses whole body: Caps.[7]

— **rigidity** of, with cough: Bell., Cina, *Cupr.*, IPEC., Led., Mosch. —2.

— — dry, spasmodic cough, preceded by rigidity of body and unconsciousness: Cina.[10]

— **trembling** of, with cough: Bell., Phos.[7]

**BREAKFAST**, cough amel. after: Aspar, Lach.—5.

**BREAST**, stitches in left, when coughing, disturbing sleep: Con.[6]

— See Mammæ.

**BREATH**, **deficient** (short) being, agg. cough: *Aur.*, Euph.[6]

— fetid: All. sat.[28]

— **gasping** for, violent spasmodic cough, commencing with gasping for breath, and continuing with repeated crowing inspirations until patient grows purple or black in face and is quite exhausted: *Coral.*[2]

— **short** before the cough: Phos.

**BREATHING**, **interrupted** (dyspnœa, etc.), with cough: Ailan., Aralia,[10] Coc. c., Ferr.,[5] Guai.,[6] Mur. ac.[7] (See p. 11.)

— **arrested**: Guai.[6] (See Chest, Obstruction of, Supplement.)

— **arrest** of, contractive pain in hypochondria with cough, arresting the breathing; the cough is prevented by the pain, unless he presses with his hand on the pit of the stomach: *Dros.*[6]

— **asthmatic** attacks at night, with slow difficult breathing, which promotes cough; Coloc.[7]

**BREATHING**, **asthma**, cough with, evening, in bed; each cough produced a stitch in scrobiculus: Amm. c.[7]

— **asthma** increased, and following a bloody night cough: Ferr. ac.[6]

— — evening when lying in bed; the cough is excited by deep inspiration: Graph.[6]

— — asthma, worse lying down, spasmodic cough; spasm of larynx; pulsations in chest; often with profuse, purulent sputa: Sil.[7]

— — asthma, worse at night, with red face; coughing spells or sensation of adhesion of lungs: Thuja.[7]

— cough which makes the boy quite breathless day and night: *Natr. m.*[6]

— **ceases**, whooping cough, child gets stiff, breathing ceases, spasmodic twitchings, after awhile consciousness returns, they vomit and recover, but slowly: Cupr.[7]

— **constriction**, sensation of, impedes respiration, with dry, tickling cough: Brom.[7]

— **difficult**, frequent cough on account of mucus in chest; mucus rises into throat, causes difficult breathing, and finally cough with expectoration: Asar.[7]

— **dyspnœa**, severe on lying down; exhaustion worse in chest after every exertion; sudden weakness, tottering while walking; blood seems to rush into chest as if it would burst; with frothy, white sputa and much retching; an hour after, slight coughing brings up gray, lumpy mucus; relieved by bending body forward: Spong.[7]

— — great dyspnœa and great anxiety but not restless; cough in violent spells, watery, profuse expectoration: Carbo v.[7]

**BREATHING**, dyspnœa (sensation of spasmodic contraction when coughing or sneezing): Merc.[7]

— **expire**, rough, scraping dry sensation in fauces, causing hacking cough, yellow mucus expectoration and hoarseness, so that he can only speak with great exertion, in a deep bass voice, together with oppression of chest, as if air were withheld on talking, coughing, so that breath could not be expired: DROS.[1]

— **inhale**, irritation to cough is so sudden and so violent can scarcely: Sepia.[6]

— **oppressed**, 6 P.M., with cough, right lung sore, sneezing: Bapt.[7]

— **sawing**, whistling, breathing between coughs: *Spong.*[7]

— **short** from rapid coughing: Dros., Euphr., Merc., Sepia.

— — short, oppressed breathing, owing to aching, rather than stitching pains, cough with green, tough expectoration and palpitation: Cann. s.[6] (Clinical.)

— — shortness of breathing, from frequent loose cough when coughing while standing: Natr. sul.[6]

— — dry cough with shortness of breath until expectoration sets in: *Guai.*[10]

— — short inspiration, long, slow expiration, epigastrium drawing fine rales, constant cough, sopor, blue face; great anguish and dread of suffocation; looks as if dying; slightly better from cold air and bending forward: Op.[7]

— **suffocated** and oppressed about 3 A.M., must sit up to get air; after cough and expectoration is better: Ant. t.[7]

**BREATHING**, **suffocation**, titillating itching in region of pit of throat, threatening suffocation, until a concussive cough sets in, continuing for hours, and causing a pain in abdomen and throat: Sil.[6]

— — cough, with sudden paroxysms of suffocation, on swallowing; respiration very short; obliged to catch for breath: Brom.[7]

— **suffocating** cough, the child becoming quite stiff and blue in face: *Ipec.*[6]

— — suffocating feeling even down to epigastrium, as if tough phlegm must work up with the cough; despair even to suicidal feeling; prostrate, tearful after the attack: Rumex.[7]

— — See also Suffocation and Suffocative Cough, pp. 110 and 111.

— **wheezing**, anxious, worse during inhalation, with violent laboring of abdominal muscles; whistling, sawing, between coughs: *Spong.*[7]

— **whistling** breathing at beginning of cough: Asar.[7]

— — phlegm in larynx causing rattling whistling breathing, until removed by coughing: Arg. n.[7]

— whistling, sawing, between coughs: *Spong.*[7]

**BRONCHI**, **crawling** in, dry whistling cough, evening in bed, caused by crawling below larynx, or as if in upper bronchi, with dyspnœa: Kreos.[7]

— **heat** in, hoarse, rough, hacking cough, excited by a sensation of heat and soreness in bronchia, without expectoration; worse evening: Eupat. per.[2]

— **irritation**, dry cough caused by irritation in bronchia, with sore pain: Arg.[7]

**BRONCHI, irritation**, or tickling at bifurcation of, excites cough: *Kali b.*[2]

— **oppression** at bifurcation of bronchi, better after successful cough: Caps.[7]

— **tickling**, dry teasing cough from tickling in bronchia, or even uncovering a hand, with tearing pain in chest, stitches, profuse sweat, and pain in stomach; cough agg. evening, and before midnight, or in morning soon after waking: Rhus.[7]

— **tickling** in, excites cough: Sepia, Sticta.—7.

**BRONCHIAL** cough, continual, deep, without pain, worse from exercise: Ailan.[7]

— dry bronchial cough, with sensation of roughness, and slight increase of heat in trachea and bronchia: Phyt.[6]

— tickling in the lower parts of the bronchial tubes, inducing cough, with slight expectoration: *Verat.*[6]

**CATARRH**, at first dry, later loose, with rattling cough, profuse sweat, sickly look, hollow eyes, restless sleep; expectoration yellow: Arg. n.[7]

— with cough: Spig.[7]

— — —, hoarseness, fluent coryza and sore throat: *Merc.*[10]

— and coryza, with feeling of exhaustion, headache, tickling roughness exciting cough: Graph.[6]

— Compare with Coryza, etc., pp. 28 and 81.

**CHEST**. See Lungs and Bronchi.

— **aching** pain in, when coughing: Mag. m., Phyt. (r.), Samb.,[6] Stront.—6.

— — pain in chest and sides, with cough: Phyt.[7]

**CHEST, aching** in side of, almost like a stinging when coughing: Coff.[6]

— **blood**, determination of blood to chest, excites dry cough: Aloe.

— **burning** in, causes dry cough: Caust.[1]

— **burning** in, with or from cough: Ailan.[7]

— — — fatiguing cough with asthma and: Carbo v.[6]

— — — up to throat with, until the expectoration becomes loose: Nitrum.[6]

— — —, discharge of blood during dry cough, with burning and soreness in chest, morning and evening: Zinc.[6]

— — — coughing excites much burning in chest, which soon passes off, until another coughing spell: *Mag. s.*[14]

— — — burning soreness in right chest; loose cough but no sputum; coldness between scapulæ: Natr. c.[7]

— **burst**, spasmodic cough, which seems as if it would: Lact. v.[2]

— **concussion** as from a shock in temples and at same time in chest, when coughing: *Lyc.*[6]

— — of whole chest from dry cough: Senega.[6]

— — See Shaking, Shocks of.

— **constriction** across, causes convulsive cough: Ipec.

— — dry cough, with constriction of chest, from tickling in throat: Lact.[2]

— — dry cough from hot constriction of chest: Carbo v.

— — cough causing sensation of constriction of chest, has to press hand on it: Dros.[10]

— — sensation of constriction impedes respiration, with dry tickling cough: Brom.[7]

**CHEST**, **constriction**, sensation as of, excites spasmodic cough: Mosch.,[2] Stram.[2]

— — constriction of upper part of chest, accompanied by soreness, when coughing: Cham.[6]

— **contraction**, sensation of painful spasmodic contraction in chest, with short asthmatic cough, excited by irritation in larynx: Amm. c.[7]

— — hoarse ringing short cough, excited by tickling in trachea, with asthmatic feeling therein; spasmodic contraction of thorax and accumulation of stringy mucus: Asaf.[7]

— — of chest with suffocating contraction of chest: Stram.[7]

— **cramp**, suffocating and choking cough, 5 A. M., as from dryness in larynx; can't speak on account of cramp in chest; red face and sweat over whole body: *Kali c.*[1]

— **crawling** and tickling in middle of chest, excites a dry convulsive cough: Kreos.

— **creeping** in, spasmodic cough from: Squil.[2]

— **cutting** in, with cough: Mag. s.[6]

— **deep**, cough deep from chest: Petr.,[7] Selen., Spong.—6.

— — dry cough at night coming deep from chest, caused by a scratching in throat, with stitches under the sternum: Petro.[7]

— — constant cough from a deep spot in chest, where he feels a pain, as if that part of the chest had become sore and bleeding from the cough: Spong.[6]

— — whooping cough, violent, racking; seemingly coming deep from chest, in paroxysms of long continuance; followed by expectoration of ropy mucus, adhering to pharynx; Lobel.[7]

**CHEST**, **empty**, continual slight cough, with feeling of hollowness and emptiness in chest after expectorating little lumps of mucus: Calad.[6]

— **excoriated**, pain as if, from coughing: Digit.[6]

— **fatigue**, evening cough fatiguing the chest, caused by an irritation in lower part of trachea: Petro.[6]

— **fly to pieces**, pains as if it would: Mur. ac.,[6] Sulph.[7]

— **hard**, oppression of chest and feeling as of a hard mass there; hard racking cough, excited by inspiration: *Sticta.*[7]

— — painful sensation, as of something hard lodged in chest; has to try to cough it up; coughing does not remove the sensation, only aggravates; water-brash often follows the cough: *Abies nigra.*

— — painful sensation, as if a stone were pressing down in pleural cavities, and violent pressure from sternum to scapulæ: Coral.[6]

— **heat** on lower part of chest and upper arm, with cough and stitches in chest: Calc. ph.[7]

— **heaviness**, weakness or soreness in chest, with dry cough: Psor.[7]

— **hollow** and empty feeling (See above under Empty): Calad.[6]

— — and cold sensation in, after expectoration: Zinc.[6]

— **hot**, whooping cough, even of sucklings, sounding like croup; rattling breathing, anxious and restless; chest and abdomen hot, urine pale: Asaf.[7]

— **infiltration** in lower part of chest causes a dry cough: Kreos.

**CHEST**, **irritation** in, excites (or agg.) cough: Ant. ox.,[1] Ars., Grat., Ph. ac., Puls., *Rhus*.—12.

— — — cough from irritation in chest, with expectoration in morning of dark blood, or of a thin, yellow, blood-streaked mucus, generally of sourish taste: Sul. ac.[10]

— — — paroxysms of asthmatic whooping cough from: Sul. ac.[2]

— **itching** in lungs low down, and extending up through trachea to nose, and itching of tip of nose, before coughing: Iod.

— — — chest (trachea), and pit of throat, with dry cough, which does not alleviate the itching: Phos.[6]

— — — spasmodic, shattering whooping cough: Puls.[2]

— — — violent spasmodic nightly (whooping) cough from: Con.[2]

— **moving** the, excites cough: Anac., Bar., Cocc., CHINA, Dros., *Lach*., Mang., Merc., Mur. ac., Natr. m., NUX V., *Phos*., Sil., STANN.—11.

— **mucus** in, cough as from mucus in chest which cannot be detached: Kreos.[6]

— — frequent cough on account of mucus in chest; mucus rises into throat, causing difficult breathing and finally cough with expectoration: Asarum.[7]

— — cough (dry, barking, hollow, croupy; wheezing, asthmatic); caused by burning tickling in larynx like a plug or valve, or by feeling of accumulation of mucus and weight in chest: Spong.[7]

— — hollow cough, especially night and morning, with tightly adherent mucus in chest, and with

**CHEST**, **mucus**—(*Continued*).

sensation of soreness on the chest: Caust.[7]

— — attacks of cough like whooping cough, during day, from mucus in chest: *Euphr*.[2]

— **obstruction**, she is suddenly attacked with a sensation of obstruction in chest, in region of heart, as if breathing would be arrested; the attacks are frequent, occur even at night when asleep, and excite an almost entirely dry cough, returning until expectoration sets in: Guai.[6]

— **narrowness** of, a single cough from feeling of: Lobel.[7]

— **oppression** of, with cough: Ailan., Apoc. c., Lac. can.,[26] Mill-lef., Nitrum,[6] Sticta.—7.

— — — after coughing: Cocc.

— — — causes deep dry cough, on inspiration, with soreness of chest: Hepar.[6]

— — — dry exhausting cough, morning and evening, with oppression of chest and roughness in throat: Rhodo.[10]

— — — and feeling of hard mass there; hard, racking cough, excited by inspiration: Sticta.[7]

— — — subdued cough from oppression of the chest: *Hepar*.[6]

— **pain** in, on coughing: Ailan.,[7] Cham.,[7] Mezer., Mosch., Naja, Psor.,[7] Samb.,[6] Upa.—1.

— —, aching in, on coughing: China, Mang., Mag. m., Phyto.,[7] Raph.,[19] Samb.,[6] Stront.—6.

— — deep in: Graph.[6]

— — dull pain in: Zinc.[7]

— — excites cough: Iod.

— — excites a hollow, deep, spasmodic cough: Meph.[10]

— — frequent dry cough with racking pains in middle of lower por-

**CHEST**, **pain**—(*Continued*).
tion of breast, extending as far as liver: Cimex.[6]
— — in left side: Sticta,[7] Tarent.[1]
— — in right side: Apis,[1] Caust., Spong., Sulph.[1]—7.
— — in right side, worse from motion, laughing, coughing, with sweat: Psor.[7]
— — in right side; must lie quietly on back and press with hand; short breath and slight cough: Cimic.[7]
— — dry teasing cough from tickling in bronchia, or uncovering even a hand, with tearing pains in chest, stitches, profuse sweat, and pain in stomach; cough is agg. evening, before midnight, or in morning soon after waking: Rhus.[7]
— — and soreness in, with dread of cough: Mag. m.[6]
— **pressure**, hoarseness, sudden and violent, with dry cough and pressure on chest, during rough weather: Mag. m.[6]
— — in, cough from: Iod.
— — great pressure on chest, when attempting to cough; this is prevented by the pain: Sil.[6]
— — painful cough, with sensation as if a stone were pressing downward in pleural cavities, and violent pressure from sternum to scapulæ: Coral.[6]
— — as from a load: Asaf., Samb. —2.
— **pressure** on (support or hold) chest, amel. pains of cough: *Arn.*,[10] *Bry.*, Cimic., Merc., *Sepia.*[10]—6.
— — on chest and groin, amel.: Borax.[5]
— — cough causing sensation of constriction of chest, has to press hand on it: Dros.[10]

**CHEST**, **pressure**, cough with pain in sternum and chest, compelling, pressure of hand on it: Kreos.[10]
— — stitches in chest (left side), worse from motion, better from external pressure: Arn.[10]
— **rattling** in, when coughing: Coc. c.,[10] Cupr.,[2] Hepar,[2] Hydr. ac.,[2] Iod.,[6] Mur. ac., Natr. c., Natr. m., Sarsap., Sulph.—7.
— — See under Respiration.—Supplement.
— — rough cough, with rattling in chest, followed by cramp in stomach, short breath, with heat in face: Mur. ac.[7]
— **raw**, pain as if from cough: Calc., *Phos.*, Sepia.—6.
— **rawness** under clavicles: Rumex.[7]
— — and soreness, with nightly heat and sweat: Zinc.[6]
— **rumbling**, after attacks of whooping cough audible rumbling, gurgling down chest: Mur. ac.[7]
— **scraping** in, with cough: Mur. ac.[6]
— **scratching**, roughness and tickling in, excite a hollow or whistling, spasmodic cough: Kreos.[2]
— **sensation** as if something in chest prevented exhalation, when talking or coughing: Dros.[7]
— **shattering**, as from shock, in chest, and also at same time in temples: Lyc.[6]
— **shootings**, cough with dyspnœa, vertigo, pains in loins and shootings in chest: Kali b.[10]
— **side** of. See under Stitches.
— **sore** pain in, when coughing; she dreads coughing, although it relieves her: Mag. m.[6]

**CHEST**, **soreness** in, from coughing: Psor.,[7] Zinc.[6]

— — cough with soreness, afterward coryza with obstruction and itching of nose: Nitrum.[6]

— — cough produces a painful soreness of chest; patient supports it with his hand when coughing: Eupat. per.[2]

— — dry cough, with soreness in chest and coryza: Gels.[10]

— — scraping cough, with soreness in whole chest and alternate hoarseness, heat and burning of hands and soles, bruised feeling of limbs, want of appetite, nausea, heat, profuse sweat at night, no thirst, constipation: Natr. c.[6]

— — cough causing sore pain in chest; pain was so bad had to go down on hands and knees, as in that position pain was eased: Eup. per. (E. W. B.)[26]

— — cough, with soreness of upper part of chest: Apis.[7]

— **spasm** of, dry titillating cough, night, with sort of: Sepia.[6]

— — — suffocative, deep, hollow whooping excited by: Samb.[2]

— **sternal** region. See Sternum, p. 105.

— **sticking** in, causes cough: Iod.

— sticking pain in left side of, with cough and want of breath when moving about, 1 P.M., relieved during rest: Natr. sul.[1]

— **stitches** in, on coughing: Calc. ph., Berb., Kali c. (r.), Kreos. (l.).[6]—7.

— — — Compare Stitches in Chest, p. 23, with Side, Stitches in, p. 20.

— — — easily angered, from it cough and stitches in chest: Arg. n.[7]

**CHEST**, **stitches** in, left side, agg. by cough, with oppression of breathing; worse from motion, better from external pressure: *Arn.*[10]

— — — left side, when breathing and coughing, pains amel. from external pressure: *Sepia.*[10]

— — — and heat on lower part of chest and upper arm, with cough: Calc. ph.[7]

— — — with cough, profuse general perspiration and pains in abdomen: Rhus.[10]

— — — cough with drawing stitches in lowest rib, near the spine: Arg.[7]

— **stuffed** sensation, cough from a: Guai.

— **stone** pressing down. See under Pressure.

— **tension** in, region of left short ribs, during cough: Hell.[7]

— — over thorax with cough: Mezer.[7]

— — in, hacking cough from: Thuja.

— **tickling** in, excites cough: Carbo an. (r.), Sarsap.—7.

— — — causes a spasmodic, shattering cough: Rhus.[2]

— — — on entering room: Nitrum.[6]

— — — hollow, spasmodic cough from: Phos.[7]

— — — low down, dry cough: PH. AC.

— — — middle of, causes dry cough: Kreos.

— — — — — causes dry hollow cough, during rest: Euph.[6]

— — itching in, causes a hollow, hacking, spasmodic, tickling cough: Phos.[2]

— — — — causes powerful, spasmodic nocturnal paroxysms of whooping cough: Con.[2]

**CHEST**, tickling itching, upper anterior part of, dry cough: Polyg.
— — — — violent racking cough; worse at night, as if head and chest would burst, sometimes with vomiturition; in two paroxysms, from tickling in larynx and upper part of chest; only by day or only by night: *Merc.*[7]
— — in, **whooping** cough from: Merc., Mur. ac.,[7] Rhus.—10.
— **tightness** in the chest, cough from tickling in throat, with a feeling of: Lact. v.[10]
— — dry tickling cough, with tightness across chest: Phos.[7]
— — dry cough, with increased tightness of chest and roughness in throat: Rhodo.[6]
— — now in left chest, now in left hypochondrium, causing hacking cough: Thuja.
— **tingling** in, excites cough: Sepia,[6] Squil.[10]
— **ulcerative** pain in, when coughing, evening and night: Mag. m.[6]
— — sensation: Nitr. ac.[6]
— **weak** feeling in, after cough: Ruta.[7]
— — — in chest, from talking, coughing or sitting too long; relieved by walking: Ph. ac.[7]
— **weakness**, dry cough, with weakness, heaviness, or soreness in chest: Psor.[7]
— **weight**, cough excited by feeling of accumulation of mucus and weight in chest: Spong.[7]
— — hard cough with weight and soreness in chest and copious expectoration: Kali b.[6]

**CHILL**, after the chill, thirst, drinking causes violent headache and dry continuous cough, from tickling in larynx, oppression of breathing: Cimex.[7]

**CHILL**, stitches in side, with troublesome cough, during chilly stage of fever, or after midnight: China.[6]
— followed by headache and cough, agg. by heat: Apis. (Deuel.)
— chilly, evening, upper arms, thighs, with thirst, drinking causes cough: Psor.[7]

**CHOKING** cough: Lyc., Sulph.
— — See Suffocative Cough, p. 110.
— — as soon as he falls into a sound sleep, has a choking cough, as if he would choke: *Lach.*[14]

**COLD** air. See p. 4.
— — going from warm to cold air: Verat. v.[7]
— **drinks.** See Water, p. 138.
— on becoming, cough: Guarea.
— **water.** See Water, p. 138.

**COLIC**, with dry, rough cough: Hepar.[7]

**COMPLAINING**, with cough: Lach.[8]
— about belly-ache before cough begins: Ph. ac.[8]

**CONCUSSIVE** cough: Secale.[7]
— — in the night, the breathing being frequently arrested, as in whooping cough, accompanied by stitches in chest, sore throat and fever: Nitr. ac.[6]
— — See Shaking Cough, p. 92.

**CONSTANT** cough: Ars.,[7] Phos.,[1] *Squil.*,[14] Sticta.[7]
— — from a deep spot in chest, where he feels a pain, as if that part of chest had become sore and bleeding from the cough: Spong.[6]
— — a deep, dry, unceasing cough: Ars.[7]
— — whenever child is laid down, particularly at night: Sepia.

**CONSTANT** cough, almost constant, harassing, tickling cough, day or night, dry or loose: *Squil.*[14]

**CONTINUOUS** uninterrupted dry cough: Euph.[6]

— hard cough: Lyc.[5]

**CORYZA.** See under Nose, p. 81.

— cough, with soreness of chest and fluent: Gels., Meph.—10.

— cough, with violent coryza and rawness in upper part of sternum: Rumex.[10]

— cough and coryza, with sneezing, commences every morning in bed before getting up and lasts until 9 A. M.: Sepia.[7]

**CROUPY** cough: Apis, Gels., Sticta.—7.

— croup, with sopor, stertorous breathing and wheezing, with open mouth and head thrown back; the child starts up, kicks about, is on point of suffocating, turns black and blue in face, after which cough with rattling breathing set in again; suffocation and paralysis of lungs appear unavoidable: Samb.[6] (Clinical.)

**CROWING** cough: Ars.[12]

**CRYING** out: Ant. t., Arn., Bell., Cham., China, Ipec., Osm., Ph. ac., Samb., Sepia, Verat.—8.

— — about belly-ache before cough begins: Ph. ac.[8]

**CYANOSIS** with cough: Verat.[7]

— See Suffocating, etc.

**DAMP** room, cough in: Bry.[2]

— See Vaults, etc.

**DAY**, cough during: Nicc.[6]

— **amel.** of dry cough, by expectoration: Guai.[1]

— — of dry cough on lying down: Sepia.[1]

— cough from 6 A. M. to 6 P. M.: Calc. ph.[7]

**DAY**, cough during, with copious, greenish, salty expectoration; worse in morning: Stann.[10]

— and night, cough with tension over the chest: Mezer.[10]

— **lying**, a dry, barking cough, from tickling in larynx and pit of stomach; worse at night, also in day when lying down: Nitr. ac.[7]

— — dry cough during day, obliging one to lie down, and ceasing while lying; cough ceased during night while lying but he had bad coryza: Sepia.[6]

— **only** during the: *Amm. c.*,[11] Arg.,[1] EUPHR.,[10] *Lach.*,[10] Merc.,[10] Nitr. ac.,[6] *Phos.*,[11] Sepia,[10] Staph.,[7] Thuja,[10] Viola od.[7]

— — cough on rising in morning, continuing until lying down again: Euphr.[7]

— — cough only at night or only by day: Merc.[7]

— — cough only during day; or cough which wakens at night: Sepia.[10]

— — cough only during day; or in morning (after rising) and evening after lying down: Thuja.[10]

— — cough by day chiefly, in long lasting spells, dry, short, violent, with much dyspnœa: Viola od.[7]

— while **sleeping**, constant waking from sense of suffocation, so that he was obliged to sit upright and cough, during the midday sleep (apparently caused by mucus in posterior nares): Euphr.[1]

**DEBILITY.** See Exhaustion.

**DEEP**, dry unceasing cough: Ars.[7]

— dull cough with bloody expectoration: Sabad.[6]

— hollow cough, toward noon, with discharge of pure blood: Sil.[6]

— cough from tickling in throat: Ambra.[1]

**DIARRHŒA**, circumscribed redness of cheeks, pain in chest, cough, coryza, then diarrhœa: *Sang.*[10]
— dry cough amel. by frequent stools: Bufo.[1]
**DINNER**, dry cough after: Kali b., Nux v.[10]
— dry cough amel. at night and after: Bar.[6]
— cough after, with soreness of throat: Phos.[6]
**DIZZINESS**, staggering after coughing: Led.[7]
**DRAUGHT**, cough worse from any, warm or cold: Caps.[7]
**DRINKING**, cough after: Carbo v.[7]
— amel. cough: Brom.[7]
— — dry cough day and night, with a burning in chest, as if something hot were there; cough diminishes after eating and drinking: *Spong.*[6]
— cough from: Kali c.[7]
— — — **beer**: Nux v.[7]
— — — **brandy**: Ferr.[7]
— — — **cold** fluids: Ars.,[12] *Thuja.*[7]
— — — **milk**: Ant. cr.
— **warm** fluids, agg. cough: Caps., Ign.—7.
— **water**. See Water, p. 138.
— **wine**. See p. 146.
**DROWSY**, with cough: Kreos.,[6] Op.[7]
**DRUNKARDS**, cough of: *Stram.*[9]
**DRY** cough: Verat. v.[7]
— or hacking cough agg. by close air or dust: Natr. ars.[1]
— cough in **bed**, evening: Agnus, Bals.,[1] Carbo v.,[6] Sepia, Stann. —7.
— — especially evening in bed till midnight, frequently with nausea and vomiting: Sepia.[10]
— — **blood**, discharge of, during dry cough, with burning and sore pain in chest, morning and evening: Zinc.[6]
**DRY** cough, ends in raising black blood: Elaps.[10]
— — **breath**, dry cough with sudden loss of: *Nux m.*[7]
— — — dry cough with difficult breathing: Lach. (Hg.)
— **cachectic** cough: Bor.[7]
— cough, **chest**, dry cough with heaviness, weakness or soreness in chest: Psor.[7]
— — — dry cough, evening and night, with burning and soreness in chest: Mag. m.[6]
— — — with rattling in: Natr. m.[7]
— — — irritation to, from scraping and crawling in chest: Con.[1]
— — **coming** and going very rapidly: Kali c.[6]
— — during **coryza**: Bell., Graph., Nitr. ac.—10.
— — **cry**, dry cough with yawning and sudden loud cry, following the paroxysms of cough: Op.[6]
— — **day** and night, expectoration copious only in the morning: Euph.[10]
— — after **dinner**: Kali b., Nux v.—10.
— — from **eating**: Æthusa.[7]
— — **evening**, agg. in: Sticta.[7]
— — — with dry cough and evening fever, he worries his family with his bad humor: Psor.[8]
— — — in bed: Agnus, Carbo v.,[6] Sepia, Stann.—7.
— — — — — agg.: Sepia.[7]
— — — — — till midnight: Sepia, Stann.—10.
— — **expectoration**, dry cough, hawking, later copious green sputum: Kali iod.[7]
— — with **hoarseness**, soreness in chest; excited by tickling in throat-pit: Sil.[7]
— — **irritation** to: Asaf.[7]
— — **larynx**, dry spot in larynx,

**DRY** cough—(*Continued*).
where there is crawling and almost constant irritation to a dry cough: Con.[1]
— — **larynx**, from irritation in, at night: Stront.[10]
— — — from itching tickling in back part of top of, evening after lying down: Bell.[7]
— — — **tickling** in, from: Bar., Carbo an., Colch., Hydras., Iod., Ipec., Nux v., Phyt., Ratan.,[10] Senega,[10] Zing.—6 and 7.
— — — — morning: *Iod.*[10]
— — — — dry cough, for three days, caused by tickling in larynx and in præcordial region, which abates only at night and after dinner: Bar.[6]
— — — — dry shaking cough from tickling in larynx, especially in open air: Senega.[10]
— — — — dry cough, with tickling in larynx and great soreness in chest: Ratan.[10]
— — — — dry barking from tickling in larynx and pit of stomach; worse at night, also in day when lying down: Nitr. ac.[7]
— — — — dry fatiguing cough from titillating in larynx; worse after midnight and in morning, and after eating, causing pain in stomach and soreness in abdominal walls: *Nux v.*[7]
— — — — evening after lying down, tingling and tickling in larynx, and dry, short, hacking cough: Caps.[6]
— — **lying** down, evening: *Bell.*, Carbo v.[6]
— — — less when sitting: Cinnab.[6]
— — **midnight**, dry cough evening in bed till: Sepia, Stann.—10.
— — **morning**: Hyper.,[7] Iod., Puls.—10.
— — — early in: Ol. an.[6]

**DRY** cough, **morning**, from tickling in larynx: Iod.[10]
— — — dry cough and prostration in morning: Hyper.[7]
— — — mostly in morning, with retching and desire to vomit and sensation as if stomach were turned inside out: Puls.[10]
— — — after rising. See under Rising, p. 36.
— — — throat, cough dry in morning, loose in afternoon, from tickling in throat, with stitches in chest: Amm. m.[7]
— — **night**: Grat., Natr. sul.,[6] Op.—7.
— — — agg. at, with gagging: Hell.[7]
— — **pharynx**, from tickling high in, evening: Carbn. s.[1]
— — **rising**, morning after: Arn.,[6] Plumb.[7]
— — — morning on: Borax,[7] Natr. sul.[6]
— — — dry cough on rising, disappears on walking: Grat.[6]
— — **room**, agg. from heat of room: Natr. ars.
— — **sitting**, after dinner: Lach.[10]
— **spasmodic**, nervous cough (peculiar to women); periodically, every night from sunset to sunrise: Aur.[7]
— cough, from **smoking**: *Hell.*[7]
— — **sternum**, irritation midsternum causes dry, incessant cough: Mang.[7]
— — — from tickling under: Zinc.[6]
— — amel. by frequent **stools**: Bufo.[1]
— — **stooping** agg.: Spig.
— — **throat**, dry cough, with hoarseness, dryness in throat, and watery coryza: Sulph.[7]
— — — hoarseness, with coryza and dry cough from titillation in throat: Sepia.[7]

**DRY** cough, **throat**, from roughness in: Carbo v.[6]

— — — from tickling in: Amm. m., Bov.—7.

— — — — frequent dry cough from tickling in throat, with constriction of chest: Lact.[2]

— — — from tickling in, when sitting: Con.[1]

— dry **tickling** paroxysmal cough; worse at night, with gaping, drowsiness, yet cannot sleep, or with spasms of lungs and blue face: Op.[7]

— dry **titillating** cough, with a sort of spasm in chest at night: Sepia.[6]

— cough, **trachea**, tickling in. See p. 129.

— — with **vomiting**: Sepia, Puls. —10.

— — — — See p. 133.

— — from **walking** in sharp, cold air: Verat.[7]

— — whooping cough, dry cough remaining after: Caust.[7]

**DULL** cough: Acon., Calad.,[16] Dig., Ferr., Mag. c.—1.

**DYSPEPTIC** cough: Lob. s.[1]

**DYSPNŒA**, etc., with cough: Lac. can.[26] See Asthmatic Affections with Cough, pp. 7 and 172.

— cough as from: Euph.[1]

— especially in the morning, with cough and expectoration of white mucus "as tough as pitch:" Kali b.[6]

— pain in left side, when coughing, with weakness and dyspnœa: Verat.[6]

— worse after coughing, with sensation of constriction of chest or stomach: Ars.[7]

**EAR**, every coughing fit is accompanied by an aching pain in ear, as if an ulcer would open: Caps.[6]

**EATING** excites or agg. cough: Æthusa,[7] All. sat.,[28] Anac., Bar., Bufo,[1] Caps., Lyc., Mag. c., Mezer., Sang., Squil.[7]

— cough caused by constrictive sensation in larynx, especially after eating: *Puls.*[1]

— **amel.**, eating apples temporarily relieves tickling in larynx which excites hoarse dry cough, or a spasmodic cough: All. c.

— — cough, with sensation of burning in chest, all amel. by eating or drinking: *Spong.*

— — tickling in larynx: Euphr.[7]

— **cold** food agg.: Thuja.[7]

— **fish**, causes cough: Lach.[6]

— **irritating** food (salt, pepper, etc.), agg. cough: Alum.[7]

— on **lying** after eating agg. of short cough: Tereb.[1]

— **sweetmeats**, cough from: Spong.

— **vinegar**: Alum.[7]

— **warm** food, amel.: Spong.[7]

**EMOTION**, or excitement agg. cough: Bufo,[1] Dros.[6]

**EPIGASTRIUM.** See Stomach-pit, p. 108.

**ERUCTATION.** See Belching. Supplement.

**EVENING** cough: Apoc. c.[7]

— — 6–7 P. M.: Ipec.

— — 7 or 8 P. M.: Sin. n.

— — 8–9 P. M.: Sepia.

— — 8.20 P. M.: Coca.

— — 9 P. M.: Lyc.

— — 11 P. M.: barking cough: Bell.[7]

— — 11 P. M.: Ant. t., Rumex, Rhus, Verat.

— — 11.30 P. M., from tickling in larynx: Coc. c.

— — cough about 9 P. M. with fever, followed by burning heat of head, cramps in legs and feet, hands and arms, and rapid pulse: Lyc.[1]

**EVENING** cough, **chest**, cough in evening, with pain in chest, caused by speaking, better from quiet: Psor.[7]
— — in **bed**, a titillating cough, violent: Lach.[6]
— **dry** cough in: Ol. an.
— — — agg. in: Sticta.[7]
— — — agg. lying down: *Ars.*
— — — in bed: Agnus, Bals., Carbo v., Sepia.
— — — — See under Dry Cough, p. 33, and Supplement, p 181.
— — — expectoration in morning, dry cough in evening: Ferr.[10]
— — — after lying down: *Bell.*
— — — severe, the morning cough, even with difficult expectoration, is much more severe and causes more pain than the dry evening cough: Squil.[10]
— — **expectoration**, cough every evening, not ceasing until he coughs up a little phlegm: *Sepia.*[7]
— — — expectoration in morning, dry cough in evening: Ferr.[10]
— — **hacking**: Ol. an.[6]
— — **hard**, dry evening cough, with pain in distant parts: Caps.[7]
— — **head**, dry short cough in evening, with subsequent weakness in head: Bar. c.[6]
— — on **lying** down. See under Lying.
— — till **midnight**: Bar., Carbo v., Caust.,[7] Ferr., Led., Mezer.,[7] Nitr. ac., Phos.,[1] *Puls.*, Rhus, Sepia, Stann., Verat.,[7] Zinc.—2.
— — **racking**, violent racking cough evening, on going to sleep, as if chest and head would fly to pieces; cough is followed by violent stretching; Merc.[6]
— — **rising** in the evening, during a violent fit of coughing, he throws up a sweet substance: Calc.[6]

**EVENING** cough, **smoking**, evening cough which makes his accustomed tobacco smoke intolerable: Arg. n.[7]
— — **vomiting**, cough in evening, with vomiting of ingesta: Rhus.[10]
— — — rising in evening, during a violent fit of coughing, he throws up a sweet substance: Calc.[6]

**EXERTION**, excites cough: Ailan., Brom., Sulph.—7.
— cough from violent exercise: Ox. ac., Verat.—7.
— violent cough and painful stitches in trachea from slightest exertion: Manc.[7]

**EXHAUSTING** cough (fatiguing, wearying): Brom.,[6] Coc. c.[7] Puls.
— — evening: Rhodo.[10]
— — — fatiguing cough with asthma and burning in chest: Carbo v.[6]
— — fatiguing cough from oppression of chest: Cocc.[6]
— — fatiguing cough, so violent she loses her senses: Kali c.[6]
— — fatiguing cough in old people; copious sputa, thick, or white: Kreos.[7]
— — violent attack of exhausting dry cough, relieved by laying hand on pit of stomach: Croc.[7]

**EXHAUSTION** after or from cough: Alum., Ant. t., *Iod.*, Kali c.[6]—2.
— — general debility, weariness and weakness of limbs: Kali b.[2]

**EXPECTORATION**, cough amel. by: Calc., Carbo an., Guai., Iod., Kreos., Lobel., Nitrum, Sepia, Zinc.—5.
— cough until raises freely, then better: Ailan.[7]
— of ropy mucus, inclining to adhere to pharynx, amel. of cough from: *Lobel.*[5]

**EXPECTORATION** of quantities of mucus from larynx and trachea amel. cough: *Iod.*[5]
**EXPIRATION** excites cough: Cann. s.[5]
— cannot expire: *Meph.*[7]
**EYE**, hæmorrhage from, from coughing: Carbo v., Cham., Nux v.[7]—2.
— lachrymation with cough: Graph., Op.
— luminous appearances before eyes, when coughing or sneezing: Kali chl.[6]
— sparks rush out of eyes when coughing: Kali c.[6]
— cough with stitches over one eye, headache and burning dryness in throat: Phos.[7]
**FACE**, perspiration on: Dros.[7]
— — profuse sweat; it stands out on face in beads; stitches in side, cough during the attack: Arg. n.[7]
— red: Graph.[7]
**FAUCES**, choking constriction in upper part of, with difficult breathing and irritable cough, or disposition to cough: Cocc.[6]
**FEET**, cough, with profuse saltish expectoration, emaciation, dyspnœa, œdema of: Samb.[6] (Clinical.)
**FEVER**, cough and hæmoptysis after typhus fever: Sul. ac.[7]
— tight dry cough, with burning fever and red face: Guai.[7]
**FOREHEAD**, bursting pain in forehead and shocks or beating as of hammers: *Natr. m.*[7]
— after the cough, pain and swimming in forehead, almost causing him to fall: Kali b.[6]
— splitting frontal pain: Sticta.[7]
— stitches in: Mezer.[6]
**FORENOON**, 10 A. M.: Aqu. petr.
— irritation in region of thyroid gland, with a good deal of coughing: Mag. c.[6]
**GAGGING**, dry cough with: Apis.[7]
— coughing and gagging: *Lyssin.* (Hg.)
— persistent cough from tickling in throat, under sternum, or in stomach; worse falling asleep or during day: Lach.[7]
**GAPING**, heat then: Sang.[7]
— cough, with sneezing, gaping, and flatulent eructations: Lobel.[7]
— dry, tickling, paroxysmal cough; worse at night, with gaping, drowsiness yet cannot sleep: Op.[7]
— See Yawning, p. 135.
**GRASPING** at genitals, spasmodic cough: Zinc.[7]
**HACKING** cough: Verat. v.[7]
— — Compare with Dry Cough.
— — from inhaling cold air: *All. c.*[7]
— — from irritation in larynx: Stront.[6]
— — from tickling in larynx: Phyto., Zing.—7.
— — — dry hacking cough, with hawking; from tickling in larynx or dryness in pharynx; worse at night as soon as he lies down: Phyto.[7]
— — in morning: Ol. an.[6]
— — agg. on motion in open air: Osm.[1]
— — from dryness in pharynx: Phyto.[7]
— — **throat**, arises from pit of throat, with a cool, salty fluid, deep in throat, posteriorly: Cann. s.[7]
— — — from irritation in: Hyper.[7]
— — — — — — as from hot air: Sum.[1]
— — — from rawness in: Caust.[7]
— — **wakening** at midnight, with scraping in chest, and sensation as if stomach would turn, as if vomiting would set in: Ruta.[6]
**HÆMORRHOIDS**, suppressed: Millef.[7]

**HÆMORRHOIDS**, cough came on after disappearance of: Euphr.[7]

**HANDS** cold when coughing: Rumex.[10]

— putting out of bed causes convulsive cough: Rhus.[1]

**HARD** cough: Secale, Spig.—7.

**HEAD, aching**: Verat.[16]

— **blows**. See Shocks.

— **bursting** pain in, on coughing: Bry., Calc., Spig.

— **concussion** as from shock in temples and at same in chest: Lyc.[6]

— **congestion** to, with the cough: Anac.[16]

— **pain** in head and abdomen, when coughing: Sulph.[10]

— pain in back of head, when coughing: Ferr.[7]

— — nightly cough almost without intermission, with pain in head and in both sides of abdomen: Lyc.[6]

— — spasmodic cough causing sore pain in top of head and great weakness: Kali c.[6]

— **pressure** on top of, on coughing: Anac.[6]

— **sore** pain on top of: Kali c.[6] (See Pain.)

— **split**, pain as if it would, from cough: Phos.[7]

— **stitches** in forehead, on coughing: Anac.[6]

— **throbbing** in forehead and temples, when coughing: Hepar.[6]

— **trembling** of head, particularly when coughing: Ant. t.[7]

**HEART**, palpitation of, with cough: Agnus, Millef., Stram.[5]—7.

— cough in paroxysms, with palpitation and nose bleed, mostly in morning: Agn. c.[7]

— ebullitions from coughing blood: Millef.[7]

**HEAT**, agg. or excited by coughing: Hyper.[7]

— after cough heat, then gaping: Sang.[7]

**HICCOUGH** during and after cough, as if he would suffocate: Tabac.[7]

**HIP**, pain above left, when coughing, as if the part would burst: Caust.[6]

**HOARSE** cough: Eup. per.,[5] Secale.[7]

— — with vomiturition, evening: Cina.[6]

**HOARSENESS** with cough: Ambra, Amm. m., Con.—12.

— nightly, hoarseness with turns of dry cough, after which she throws off blood-tinged mucus mixed with saliva: Arg. n.[16]

**HOLLOW** cough, morning after rising: Cina.[6]

— — especially night and morning, with tightly adherent mucus in chest, and with sensation of soreness in chest: Caust.[7]

—, racking, spasmodic cough, from tickling in larynx; before the cough he loses his breath; after the cough, dizziness, staggering; double, sobbing inspiration: Ledum.[7]

— cough at night: Caust., Samb. —7.

— — caused by a filling up sensation in throat: Apis.[1]

**HUNGER**, cough from: Kali c.[7]

**HYSTERICAL** cough: Der.[1]

**INSPIRATION** excites cough: Camph.[7]

— after the cough, double sobbing inspiration: Led.[7]

— chest feels worse in open air, every inspiration provokes cough: Mag. m.[1]

— deep excites cough: Dulc., Kali b., Plumb.[6]—7.

**INSPIRATION**, deep, agg. dry cough, which is excited by tickling in larynx or trachea, and by external pressure, with soreness in larynx or trachea; worse at night after retiring: Bell., Lach., *Rumex*.—25.

— cough threatening suffocation, with spasmodic contraction of larynx; every inspiration increases the cough: Meng.[14]

— on forced inspiration through nose, evening, cough: Sapon.[1]

— free inhalation and impeded exhalation, with constantly increasing tickling in larynx, which compelled patient to make very energetic but unavailing efforts to cough, continue until he sinks, exhausted and covered with sweat, upon a couch, when spasm (of glottis) relaxes and he can cough and inhale freer: Chlorine (gas cured). (Dunham.)

**INSPIRING** cold air, cough on: Agnus.[7]

— inhaled air is very cold: Brom., Sulph.—2.

**IRRITATION** to cough, agg. by coughing: Squil.[2]

— — — in throat, on inspiration: Asar.[7]

— — — from sticking griping: Mur. ac.[1]

**KNEE**, shock in, causing it to give way, with pain in patella when walking: Nitr. ac.[6]

— cough only relieved by getting on hands and knees: Eup. per. (E. W. B.)

**LARYNX**, **burning** titillation in upper part of, causing cough: Euph.[6]

— **cold** sensation in, on inspiration: Brom., Sulph.—2.

— **constriction**, sudden attacks of a dry and hacking cough, as if occasioned by a spasmodic constriction of larynx, which seemed to be lined with dry mucus: Coffea.[6]

**LARYNX, constrict'n**—(*Cont'd*).

— **contractive** sensation in larynx excites cough, especially after eating, with vomiting and bleeding at nose: Puls.[6]

— — spasmodic contraction of larynx; effort at inspiration excites cough: Meny.[10]

— **crawling** (creeping) in upper part of, causing cough: Prun. sp.[6]

— — cough excited by crawling or tickling in larynx, especially during pregnancy: *Sabina*.[14]

— — dry, whistling cough, evening in bed, caused by crawling below larynx, or as if in upper bronchi, with dyspnœa: Kreos.[7]

— **dryness** extends into larynx, rendering voice husky, and often inducing cough: *Bell.*[1]

— — in, with cough: Mang.[6]

— — — — dry painful cough; Copaiba.[10]

— **irritation** to cough felt in: Ang., Asar., *Calad.*, Lith. c., Men., Merc., Ph. ac., *Rhus*, *Squil.*, Stann.—12.

— — — **asthma**, short turns of cough, from irritation in larynx, hurried breathing and asthma, or with pain in abdomen: Canth.[6]

— — — — short, asthmatic cough from irritation in larynx, with painful sensation of spasmodic contraction of chest: Amm. c.[7]

— — — **dry** cough at night from irritation in larynx: Stront.[10]

— — — **evening**, violent irritation inducing cough, evening in bed and in morning; the irritation is too deep in larynx to be reached by the cough, hence its violence and inability to detach the phlegm: Mezer.[6]

**LARYNX, irritation** to cough felt in upper part of: Cocc., Euph.—6.

— — — violent irritation in upper part of larynx, causing short, hacking cough: Euph.[6]

— **itching** in, intolerable itching titillation in larynx, obliging one to cough violently: Lyc.[6]

— **moisture** in, cough as if caused by increase of: Lach.[6] (Clinical.)

— **mucus**, laughing produces mucus in, and excites cough: Arg.[10]

— — phlegm in, causing rattling, whistling breathing until removed in small lumps by coughing: Arg. n.[7]

— — constant hawking and gagging, on account of viscid, green mucus in larynx and trachea: Paris.[7]

— **pain** in larynx and sternum, from coughing: China.[7]

— — rough pain in, during cough: Lach.[6]

— — in, so severe, the patient grasps the larynx and tries to suppress the cough: *All. c.*

— from **pressure** of clothes on: Apis, Lach.

— — See Throat, Touching, p. 122.

— **prickling** in larynx and trachea inducing hacking, with subsequent dryness of mouth and throat: Laur.[6]

— **rattling** in, when coughing much: Brom.[7]

— **raw**, a scraping, sore, raw pain in, from cough: Sepia.[6]

— **roughness** and soreness in, with cough: Phos.[7]

— **scraping** in, which excites cough, and no other symptom: All. sat.[28]

— **smoky**, cough excited by smoky sensation, or as of vapor of sul-

**LARYNX, smoky**—(*Continued*). phur, or by constant titillation in: Ars.[7]

— **soreness** in, on coughing: Caust.,[6] Kali iod.[6]

— — dry, hollow cough, five or six turns at a time, with soreness in larynx, every turn of cough causing pain, and almost arresting breathing: Caust.[6]

— **spasm** of, during cough: Hyos.[7]

— — cough threatening suffocation, with spasmodic contraction of larynx; every effort at inspiration increases the cough: Men.[14]

— **stitches** in, with the cough: Aloe (r.),[7] Dros.,[6] Kali c.[1]

— — — cough during night, with stitches in: Phos.[28]

— **swollen**, suffocative cough from: *Kali iod.*[7]

— **tickling** (see irritation) in, excites cough: *Arn.*,[7] Carbo an., Carbo v., Colch., Lyc., Phyt. (l.), Sticta.[7]—6.

— **tickling** burning in, with violent paroxysms of cough: *Bell.*

— — — — like a plug or valve, causes dry, barking, hollow, croupy; wheezing, asthmatic coughs: Spong.[7]

— — in, a cough day and night from: Arn.[7]

— — — **dry** cough from: Bar.,[6] Carbo an.,[6] Colch.,[6] Iod.,[7] Ipec.,[7] Hydras., Phyto., Nux v., Senega,[10] Zing.—7.

— — — — See also under Dry Cough. Supplement.

— — — **evening**, cough without expectoration, brought on by tickling in larynx in: Carbo an.[6]

— — — **hacking** cough from: Colch., Dros., Phyt.[7]—6.

— — — **harsh**, dry ringing cough from constant tickling in larynx; the cough causes pain in larynx

**LARYNX**, **tickling** in—(*Cont'd*). which makes the patient crouch from it, and try to support larynx and suppress the cough: *All. c.*[2]

— — — **hollow**, deep, hoarse cough with, like a trumpet, from tickling in larynx and chest: Verb.[10]

— — — **hollow**, racking, spasmodic cough from: Led.[7]

— — — **intolerable** tingling and tickling in larynx, which can only be relieved by coughing and hawking, with accumulation of mucus in mouth, early morning in bed: Iod.[6]

— **tickling itching** in, dry cough: Lyc.[6]

— — below, cough from: Prun. sp.[6]

— — in, after **midnight**, toward morning, cough from: Sepia.[7]

— — — **paroxysmal** cough from: Anth. n.,[1] Chelid.,[2] Kali c.[7]

— — — **racking**, hollow, spasmodic cough: Led.[7]

— — — —, violent cough: Merc.[7] (See Violent Cough.)

— — — **severe** cough: Phyt.[7]

— — — **short** cough: Colch.[6]

— — — — titillation in larynx inducing a short and hacking cough, with sensation as if a soft body (a feather) had lodged in larynx, with fine stitches in larynx extending down to right side of œsophagus: Dros.[6]

— — — **spasmodic** cough: All. c., Bad., Iod., Ipec., Led., Mag. c.,[7] Nitr. ac., Sepia, Sulph.—2.

— — — — spasmodic, shattering cough from tickling creeping in larynx, in trachea, and in chest: Rhus.[2]

— — — — See Spasmodic Cough, p. 101.

— — — when **smoking**, sudden violent cough from: *Euphr.*

**LARYNX**, **tickling** in, **subdued** cough from: Digit.[6]

— — at **top** of, on lying down, excites cough: Kali b.[6]

— — sensation in upper and anterior part of larynx, inclination to cough from; worse lying or from talking: Lac. can.[26]

— — in, **violent** racking cough; worse at night, as if head and chest would burst, sometimes with vomiturition; in two paroxysms, from tickling in larynx and upper part of chest: Merc.[7]

— — — while **walking** in open air, cough from; larynx feels swollen: Ox. ac.[7]

— — — **wheezing** asthmatic cough caused by burning tickling in larynx, like a plug or valve, or by a feeling of accumulation of mucus in chest: Spongia.[7]

— **tingling** in, causes cough: Caps.[6]

— **ulcerative** pain in, from cough: Carbo v.[6]

**LAUGHING**, produces mucus in larynx and excites cough: Arg. met.[10]

**LIMBS**, pains in legs: Sang.[7]

— cough about 9 P. M., with fever, followed by burning heat, cramps in legs and feet, hands and arms, and rapid pulse: Lyc.[1]

— stiffness in back and limbs: Rhus.[7]

**LIVER**, **stitches** in, when coughing: Bry., Carbo v., Eupat. per.,[7] Kali c., Merc., Natr. m.[10]—2.

— See Hypochondria, p. 2.

**LOINS**, cough in women, with pain in: Arundo.[1]

**LOOKING** at bright objects, excites cough: Stram.[7]

**LOOSE** cough: Amm. m., Sil., Squil., Sticta.—7.

**LOOSE** cough, **afternoon**, dry cough in morning (from tickling in throat), with stitches in chest or left hypochondrium, becomes loose in afternoon: Amm. m.[6]
— — **chest**, from tickling deep in: Graph.[7]
— — — soreness and pressure in chest, with: Sulph.[7]
— — — loose rattling cough with oppression of chest: Apoc. c.[7]
— — with **expectoration**: Con., Phos.—12.
— — **morning**: Meph., Natr. sul.,[6] Sticta.—7.
— — — the loose morning cough is more fatiguing than the dry evening cough: Squil.[7]
— — —, less free during day: Sticta.[7]
— — **night**: Amm. m.,[7] Eupat. per.[6]
— — — and day: Sil.[7]
— — —, with stitches in left hypochondrium, lying on back; worse when turning side, and from cold food: Amm. m.[7]
— — **throat**, from tickling in, morning; purulent sputa and pain about last ribs, left side: Natr. sul.[7]

**LOUD** barking cough, in hysteria: Verat.[7]

**LUNGS.** See Chest and Bronchi.
— itching low down in lungs and extending up through the trachea to nose, and itching of tip of nose before coughing: Iod. (Brigham.)
— pain in, from cough: Ailan. (l.)[7]
— — —, — — and dyspnœa: Zing.[10]
— — — apices of, with dry cough: Coc. c.[1]
— spasm of, commencing with desire to cough, increasing gradually, and driving one to despair: Mosch.[6]

**LYING** excites cough: Ant. t., Dulc.,[7] Phyto.[7]
— cough **agg.** as soon as one lies down: Ars., Bry., Con., *Hyos.*, Petro., *Puls.*, Sabad.
— — — worse when lying until he gets fairly settled: Caps.[7]
— **amel.**, dry cough during day, obliging one to lie down, and ceasing while lying down; cough ceased during night while lying, but he had coryza: Sepia.[6]
— in bed, excites cough: Petro.[6]
— **day**, cough worse at night and also in day when: Nitr. ac.[7]
— in **evening**, cough on: Nicc.,[10] Petro.,[6] Ph. ac.,[6] Psor., Puls., Thuja, Rumex.—7.
— — dry cough; worse evening and night, can neither sleep nor lie down: Sticta.[7]
— — **dry** cough in: *Bell.*, Borax, Ferr., Marum, Natr. m., Nicc.,[10] Nux v.
— — — dry cachectic cough, especially in morning when rising and evening when lying down, with stitching pain in right chest and flank, relieved by pressure; washing parts with cold water affords most relief: Borax.[7]
— — — in evening after lying down in bed, tickling itching sensation in back part of top of larynx, causing short, dry cough, which he cannot suppress: *Bell.*[1]
— — — dry cough evening after lying down, with expectoration after walking: Ferr.[6]
— — — dry cough from tickling in trachea, evening after lying down: Nicco.[10]
— — during the evening cough, after lying down the expectoration becomes loose; is easier when he turns from left to right side: Thuja.[10]

**LYING**, **evening hacking** cough: Caps.[6]
— — very **severe** cough: *Sepia.*
— — **violent** cough evening while lying, must rise, no expectoration; the irritation to cough is in a little spot posteriorly and inferiorly in throat: Lith. c.[7]
— — **whooping** cough: Coc. c., Dros., Natr. m.—2.
— at **night**, agg.: Amm. m.,[10] Gamb.,[6] Phyto.[7]
— — — cough on lying down at night: Dolich.[7]
— — — and in sleep cough is absent: Kali b.[6]
— on **left** side agg.: China.[7]

**MAMMÆ**, constant cough, and hawking pain in chest in mammary regions: Natr. ph.[1]
— during cough stitches in left breast, disturbing sleep: Con.[6]
— stitches in right breast, near nipple, at every turn of the cough, in evening: Borax.[6]

**MANIA**, cough with: Verat.
— coughing up phlegm, with gagging and retching in: Bell.[8]

**MEAL**, cough after a: Ars., *Bell.*, Bry., Calc., Carbo v., China, *Ferr.*, Hepar, Lach., Nux v., Phos., Puls., Sang., Sil., Staph., Sulph.—6.
— cough after every: *China.*
— — — a meal or only when walking in open air: Sulph.[10]
— See after Eating, pp. 38, 183.

**MEASLES** cough with (or after): Squil.[7]
— eruption develops, and day cough is amel.: Cupr.[7]
— nocturnal loose cough after the eruptive stage of: Eupat. per.[7]
— even with typhoid symptoms; catarrh prominent; eruption tardy; earache; ophthalmia; short, dry cough, pain in chest;

**MEASLES**—(*Continued*).
or, loose, rattling cough, which is prone to remain as sequel: Puls.[7]

**MEAT**, cough after eating: Staph.[7]

**MENSES**, cough during: Coff.[12]
— during, roughness in throat causing cough: Castor.[6]
— suppressed, cough from: Mille., Puls.—7.

**MENTAL** mood changeable before the cough, merry or melancholy: Ferr.[8]

**METALLIC** cough: Rumex.[7]

**MIDNIGHT**, cough toward, with oppression of chest and coldness: Grat.[6]
— fit of coughing about, feels as if something had gotten into her throat and would choke her: Cham.[6]
— **after**: Sepia.[7]
— — dry spasmodic cough, with vomiturition: Bell.[10]
— — dry cough, with violent beating of heart and arteries: Calc.[6]
— **before**, night in bed, cough after having slept an hour or two; occurring before midnight, awakes patient; *Aralia.*
— — barking cough, waking suddenly at 11 P. M.; face fiery red, crying out with the cough: Bell.[7]
— — hoarse barking cough, in attacks every night at 11 P. M., and at 2 and 5 A. M.: Rumex.[7]

**MOAN**, sudden and involuntary moan, half a cough and half a moan; caused by irritation in throat: Calad.[7]

**MORNING** cough; Vic.[1]
— agg. at 10–11 A. M.: Coc. c.[1]
—, early: Hepar, Nux v.
— — See after Midnight.
— — begins to cough at 3 A. M., and repeats every half hour: *Kali c.*

**MORNING**, early cough from 6 A. M. to 6 P. M.: Calc. ph.[7]
— cough **agg.** in: Thuja,[10] Verat.[7]
— cough, first barking, dry cough, followed by expectoration of viscid stringy mucus; the difficult expectoration causes retching and vomiting: Coc. c.[10]
— in **bed**: Nux v.[6]
— **dry** cough in, loose in afternoon (from tickling in throat), with stitches in chest or left hypochondrium: Amm. m.[7]
— dry in, evening cough loose: Bov.[7]
— dry cough and prostration in: Hyper.[7]
— dry spasmodic cough in, causing retching: Kreos.[7]
— cough, even with difficult expectoration is more severe and causes more pain than the dry evening cough: Squil.[10]
— **loose** cough in: Natr. sul.[6]
— **on rising**: Bor.,[7] Canth.,[6] Carbo an.,[7] Thuja.[10]
— — — cough only during day or in, on rising and in evening after lying down: Thuja.[10]
— — — violent fits of cough before retiring and on rising: Ailan.[7]
— **after rising**: Ant. cr.,[7] Carbo v.[6]
— — — dry cough: Arn., Plumb. —7.
— — — — — with asthma: Digit.[6]
— — — paroxysmal cough: Ant. cr.[7]
— — — short cough from irritation in throat: China.[6]
— — — slight cough, from tickling low in trachea, every morning after rising: Arn.[7]
— **straining** cough in the morning, straining the chest, with expectoration of mucus and blood: Selen.[10]

**MORNING**, **spasmodic**, dry, cough in, causing retching: Kreos.[7]
— **throat**, tickling in throat and cough preventing him from lying in bed: Coc. c.[1]
— **after waking**, cough worse evening and before midnight, or in morning soon after waking: Rhus.[7]
— **on waking**: Arn.,[1] Kali b.[7]
— — — agg.: Psor.[7]
— — — cough with tough, yellow phlegm on waking in the: Aurum.[7]

**MOUTH**, **bleeding** from: Arn., Bell., China, Kreos., Led., Lyc. —6.
— **closed**, scratching, rawness and bleeding in throat and a disposition to keep the jaws tightly closed: Kobalt.[7]
— rinsing, causes cough: Coc. c.[1]
— loose cough, with soreness in mouth and throat: Mag. s.[10]

**MOVEMENT**, cough agg. by least motion, especially at night: Bell.[10]
— rapid motion, cough worse from: Natr. m.[7]

**NARES**, pain in posterior, as from air passing through with violence, when coughing or talking: Mag. sul.[10]

**NERVOUS**: dry, spasmodic nervous cough, peculiar to women; periodically, every night from sunset to sunrise: Aurum.[7]
— spasmodic cough: Caps.[7]

**NIGHT** cough: Apoc. c.,[7] Castor.
— – **agg.** at: Manc., Phos.—7.
— — — cough particularly at night, with tough slimy expectoration tasting bitter: Cham.[7]
— — — much more cough at night than during day (and more in day when lying[6]); able to sleep

**NIGHT** cough **agg.**—(*Continued*).
only toward morning: Nitr. ac.[1]
— — in **bed**, before sleeping: Carbo v.[6]
— — — See Evening in Bed.
— cough all night with dull pain in chest: Zinc.[7]
— chest full of phlegm, nightly cough: Bar.[7]
— **continuous** nightly cough, almost without intermission, with pain in head and both sides of abdomen: Lyc.[6]
— **concussive** cough in the, breathing being frequently arrested, as in whooping cough, accompanied by stitches in chest, sore throat and fever: Nitr. ac.[6]
— dry cough, agg. at: Calc., Sticta. —7.
— — — agg. at, with gagging: Hell.[7]
— — — amel. on sitting up: Rhus.
— — — in bed: Calc.[10]
— — — with catarrh: Spig.[7]
— — — coming deep from chest, caused by scratching in throat: Petro.[10]
— — — evening, and night in bed during sleep; expectoration during day: Calc.[10]
— — pneumonia, with cerebral symptoms, delirium, sopor; dry, fatiguing night cough, or rattling in chest: Hyos.[7]
— — dry hard cough, evening and night, pain in distant parts: *Caps.*[10]
— — — leaves him no rest; feels hot and sweats: Sabad.[6]
— **hoarseness**, cough at, with: Calc.[6]
— **lying** down at, agg. cough: Gamb.,[6] Kali br.,[1] Phyt.[7]
— — on back, cough at, when: Amm. m.[10]

**NIGHT, lying** down, cough on, at: Dolich.[7]
— — on right side, dry cough only at night when: Carbo an.[6]
— **sit up**, violent cough at night, compelling one to sit up and hold head: Nicc.[10]
— — — nightly cough, must sit up as soon as cough commences: Ars.[7]
— **sleep**, after going to, then very severe cough: Petr.[6]
— from **sunset** to sunrise, periodic cough, every: Aur.[7]
— **sweats.** See Perspiration.
— **violent** cough at; with much scraping in larynx, pain in fauces, and thick mucus: *Cycl.*[1]
— **waking** from coughing: *Bell.*,[7] Coc. c., Coff.[6]
— — at 2 A. M.: Dros.[6]
— — from sneezing, with tickling in throat, causing cough: Amm. mur.[7]
— awakens often, all stopped up with mucus, must expectorate before he can breathe easily: Nitr. ac.[7]
— **whooping** cough, agg. at: Con.[6] (See p. 81.)

**NOISE**, a gurgling noise downward after coughing: CINA.[1]

**NOSE**, bleeding of, with cough: Agnus.[7]

**OCCIPUT**, pain in, on coughing: Lact.[6]

— stitches in: Merc. sol.[6]

**OLD** people, cough of: Alum.[7]
— See Aged, p. 4.

**PAINFUL** cough: Illicium.[7]
— cough consisting of a few, but intensely painful turns, with expectoration of small lumps of mucus (leaving an hollow, empty feeling in chest): Calad.[6]

**PANTING** cough, with oppression of chest: Phos.[6]

**PANTING** cough with audible rumbling in chest from above downward: Mur. ac.[6]

**PAROXYSMAL**, cough in sudden paroxysms convulses whole body: Caps.[7]

— dry cough in paroxysms: Chelid.[7]

— cough in paroxysms, with palpitation and nose bleed, mostly in morning: Agnus.[7]

— — from tickling in larynx: Calc.,[1] Kali c.[7]

— — — — throat, larynx, or bronchi, with dislodgment of tenacious mucus or pus, which must be swallowed: Kali c.[7]

— cough, morning: Agnus.[7]

— — agg. at night: Op.[7]

— — short cough in rapid succession of paroxysms: Coff.[6]

— — **throat**, from tickling in: Kali c.[7]

**PERIODIC** cough: Lact.[1]

—, painless, spasmodic cough in a shrill, screeching tone; worse in morning: Stram.[7]

— — from sunset to sunrise: Aur.[7]

**PERSPIRATION**, cough with profuse sweat on whole body: *Op.*[7]

— fetid sweat after coughing: Hepar.[6]

**PHARYNX**, burning in, on coughing: Mag. s., Sulph.—1.

— — — as far down as above pit of stomach, all day, with dry cough: Mag. s.[1]

— dryness and scraping in, causing a suffocative cough: Cycl.[10]

— Compare with Fauces.

— pain in, during dry cough: Mag. m.[1]

— **soreness** in from cough: Coca.[1]

— **tickling** in, excites cough: Coc. c.[1]

**PREGNANCY**, cough during, worse at night: Con.[7]

**QUIET**, amel. cough (worse from talking): Psor.[7]

**RACKING**, severe, dry cough, caused by tickling in right side of trachea, below larynx: Sticta.[7]

— violent, racking cough, worse at night, as if head and chest would burst, sometimes with vomiturition; in two paroxysms, from tickling in larynx and upper part of chest: Merc.[7]

**RASPING**, loud, violent dry cough, caused by dryness of the throat; nothing seems to affect the cough: Stram.[1]

**RATTLING** cough, disturbing sleep: Squil.[7]

— — morning: Meph.[10]

— — in spells: Cina.[7]

— dry spasmodic rattling cough, but nothing loosens: Verat.[7]

— loose, rattling cough, with some expectoration, morning: Stram.[1]

**RESPIRATION.** See also under Breathing. Supplement.

— **crepitating**: *Ant. t.*, Bell., Carbo an., Carbo v., Caust., Cupr., Hepar, Hyos., Ipec., Laur., Natr. m., Puls., Samb., Sepia, Sil., Squil.—2.

— **difficult**, relieved by cough: Acon.[1]

— **interrupted** (short, etc.), with or after cough: ALUMEN,[1] Amm. m.,[12] Aspar., China, Coc. c.,[10] Coral., *Cupr. s.*,[1] Digit., Euph.,[24] Eupion,[1] Kali c.,[2] Lact.,[10] Laur.,[2] Mur. ac.,[7] *Samb.*,[11] Sang., Sulph., Sul. ac.,[2] Viola od.[7]

— **irregular** during cough: Cupr. s.[1]

— **rattling** before coughing: Squil.[1]

— rattling: Cham., Cina, Cupr., Hepar, Hydr. ac.—2.

— **short** before cough: Lyc.[1] (See p. 12.)

**RESPIRATION, stertorous,** fine rales, constant cough, sopor, bluish face, anguish, fear of suffocation; stertorous breathing: *Opium.*[7]

— **wheezing**, with cough and frothy expectoration: ARS.[7]

— — See p. 139.

— **whistling**, during cough: Lyc.[1]

**RIBS**, painful weariness in region of last short: Puls.[6]

**RISING**, from bed, cough on: Canth.[6]

— dry cough on rising, going off on walking: Grat.[6]

— in evening, during a violent fit of coughing, he throws up a sweet substance: Calc.[6]

— See also under Morning.

**ROOM**, changing rooms, excites cough: *Rumex.*

— warm, agg. cough: Brom.[1]

**ROUGH**, shaking cough: Ipec.[7]

— cough, with pain in chest, followed by cramp in stomach; short breath; with heat in face: Mur. ac.[7]

— — with scraping under sternum: Cann. ind.[7]

— — — pain in throat: Carbo an.[6]

**SCAPULA**, cough with pain under left: Sticta.[7]

— — — stitches under left: Sulph.[7]

**SCRAPING** cough: Rhod.[6]

— — with profuse, thick, yellow or white mucous expectoration: Kreos.[7]

**SCROFULOUS** persons, cough in: Con.[6] (Clinical.)

**SHAKING** cough. Compare with Concussive Cough, p. 27.

**SHATTERING** cough: Ipec.[2] (See p. 101, 2d col.)

**SHORT** cough: Verat. v.[7]

— — **afternoon**: Anac.[6]

— — **chest**, with rattling in: Natr. c.[7]

**SHORT** cough with **expectoration** of bright, red blood, which seemingly comes from larynx: Kobalt.[7]

— — with expectoration of some mucus, especially after a meal: Caust.[6]

— — after a **meal**: Caust.[6]

— — in **morning**: Nitr. ac.[6]

— rattling cough disturbing sleep: Squil.[7]

— — **throat**, from irritation in: Carbo v.[6]

**SHRILL** cough: Ant. t.[7]

**SIGHT** dim on coughing: Coff.[6]

**SIDE**, aching pain in chest and side, with cough: Phyt.[7]

— cough causing pain in loins and sides, causing him to hold them: Kali b.[6]

— stitches in, on coughing: Crotal.,[6] Puls.[6]

— See also under Chest.

— turning from left to right side, amel. cough: Thuja.[5]

**SINGING**, excites cough: Alum., Hyos., Rumex.—7.

**SITTING**, cough did not trouble him while walking, but as soon as he sat down it returned: Canc. f.

**SITTING up** (rising up) cough when lying, amel. on rising: (1) Hyos., Mezer., *Puls.*, *Rhus*, *Sabad.*, Sulph. (2) Con., Ipec., Nitr. ac., Phos., Sepia, Sil. (Jahr's *Clinical Guide.*)

— — amel. cough: Eupion, Natr. sul., Tarent.—1.

**SLEEP**, cough worse in bed, also from sleeping: Lachn.[7]

— severe cough, especially after lying and sleeping: Apis.[7]

— cough after going to sleep: Coff.[6]

— — — See During Sleep, p. 98.

— **disturbed** by cough: Calad., Phos.,[6] Rhod.,[6] Squil., Sticta.—7.

**SLEEP, disturbed,** dry cough, worse evening and night; can neither sleep nor lie down: Sticta.[7]

— — cough fatigues, and hinders sleep: Daphne.[1]

— — hollow, mostly dry cough, with pressure in pit of stomach, hindering sleep the whole night: Phos.[6]

— — panting cough, preventing sleep: Calad.[7]

— — short, rattling cough, disturbing sleep: Squil.[7]

— **during**, violent cough during: Cham.[6]

— — the cough was usually most violent during sleep, was never caused by talking or walking, even against a cold wind: *Cylc.*[1]

— **going to**, cough after: Coff.[6]

— — — suffocative cough evening after: Aralia.

— on **going to**, violent racking cough evening when he was on the point of going to sleep, as if chest and head would fly to pieces: Merc.[6]

**SLEEPLESSNESS**, with cough: Ars., Lact.

**SLEEPY**, drowsy, with cough: Kreos., Op.—7.

— See Cough during Sleep.

**SMOKING** excites cough: Arg. n.[11]

— convulsive cough, agg. by: Lac. ac.[1]

**SNEEZING** after coughing: Anac.,[2] Lyc.[6]

— with cough: All. c., Alum., Anac., Bad., Carbo an., Carbo v., China, Cina,[7] Con., Eup. per., Hepar, Kali c., Kreos., Lobel.,[7] Merc., Nux v., Sepia, Sil., Staph. —2.

— cough with sneezing, gaping and flatulent eructations: Lobel.[7]

**SPASMODIC** cough: Cact., Caps., Coloc.[6]—7.

— — **chest**, spasmodic, tickling cough as from "down" in larynx, suprasternal fossa, and whole chest as far as epigastrium; evening without, morning with expectoration of dark blood, or of tenacious, whitish mucus of sourish, herby taste: Ph. ac.[7]

— —, child puts hands to genitals: Zinc.[7]

— — only during **day**: Staph.[7]

— — after **eating**, with vomiting of food: Ferr.[7]

— — — spasmodic cough in morning, amel. by eating: Ferr.[10]

— — **evening**, agg.: Carbo v., Stram.—6.

— — — hollow, spasmodic cough in evening from sensation of sulphur or dust in pit of throat; in the morning, from a tickling above the pit of stomach; difficult expectoration in evening, tasting and smelling like an old catarrh: Ign.[10]

— — — spasmodic cough, worse morning and evening, with darkness around the eyes: Stram.[6]

— — **larynx**, spasmodic cough from tickling in larynx; morning and day expectoration of yellow, thin or tough mucus, or of dark blood tasting salty; cough worse evening till midnight: Mag. c.[10]

— — — spasmodic cough, in short but frequently returning attacks, caused by tickling in throat and larynx; during morning and day the cough is loose, but the yellow pus and tough mucus has to be swallowed (with sore pain on top of head and subsequent great weakness[6]): Kali c.[10]

— — **midnight**, agg. after: Bell.[6]

**SPASMODIC** cough in **morning**: Ferr.,[10] Ign., Stram.—7.
— — — hollow spasmodic cough, in morning from tickling above pit of stomach; in evening from sensation as of sulphur fumes or dust in pit of throat: Ign.[7]
— — — periodical, painless spasmodic cough, in a shrill, screeching tone, worse in morning: Stram.[7]
— — **stomach**, pit of, tickling in morning: Ign.[7]
— — **throat**, from scraping and roughness in: Digit.[10] (See Trachea, roughness in, p. 103.)

**SPEAK**, attacks of cough, resembling whooping cough, whenever he begins to speak: Anac.[6]
— exhausting cough which does not permit him to speak: Brom.[6]
— cough accompanied with hawking and flow of saliva; the cough scarcely allowing one to speak: Lach.[6]

**SPEAKING** (talking) excites cough: Alum., Hyos.—7.
— cough evening with pain in chest, caused by speaking, better from quiet: Psor.[7]
— **loud**, causes cough: Ambra, Phos.—7.

**SPLEEN**, stitches in, when coughing: Bell., Carbo v., Con., Sulph., Zinc.—2.
— — See Left Hypochondria.

**STERNUM aching**. See Pain, p. 106.
— **burning** and pressure under sternum, often with cough: Asaf.[7]
— **crawling**, dry cough with tickling in throat-pit and a crawling sensation under sternum (cough becoming very severe, causing pain beneath sternum, no expectoration): SANG.[1]

**STERNUM**, **cutting** under, with cough: Nitrum.[6]
— **irritation** behind sternum causes tickling cough: Rumex.[10]
— **irritation** under, causes cough: Arg. n.[7]
— **pain** behind, when coughing: Sang.[1]
— — in, with cough: Cimex, Nitrum.[6]
— — behind mid-sternum, on coughing: Rumex.[7]
— — through mid-sternum with cough: Phyt.[7]
— — very painful cough; it seems as if a stone were lying on diaphragm; it presses downward and causes a violent pain in chest beneath sternum, thence the pain extended to scapula, it gradually subsided as cough was relieved: Coral.[1]
— **roughness** under, causing cough: Nitrum.[6]
— **scraping** under, with rough cough: Cann. ind.[7]
— **soreness** behind, on coughing: Cina,[6] Psor., Rumex.—10.
— — violent cough, as if sternum would fly to pieces; it feels sore and bruised, especially when talking, laughing, or yawning: Mur. ac.[6]
— **stitches** in on coughing: Con.,[6] Petro.[7]
— — See p. 23.
— **tickling** under, in throat, or in stomach, causes gagging, persistent cough: Lach.[7]
— — constant tickling under middle of, causing hacking cough, worse from talking or moving: Calc. (Hg.)
— — under middle of: Lac. can.[26]
— **tingling**, in centre of, cough as if caused by: Con.[6]

**STERNUM**, **ulcerative** pain behind, on coughing: Staph.[6]

**STERTOROUS** breathing with cough: Bell.,[5] *Op.*[7]

**STOMACH**, burning in, when coughing: Hepar.[6]

— **comes** from, cough seems to: Lach.[6]

— **pain** in, from cough: Lobel.[7]

— — — in pit of stomach, on coughing: Arundo,[1] Hyos.[7]

— — hard and fatiguing cough, even unto vomiting, with difficult expectoration; pain in pit of stomach, obliging one to press hand on it; cough exists only during the day, worse after sleeping or eating fish, and in damp weather, still more after walking, and particularly after talking, as if throat became dry: Lach.[6]

— **shooting**, cough with shooting in scrobiculum, leaving when sputum is raised: Zinc.[7]

— **sticking** pain in epigastrium, with cough, must press it with the hand: Phos.[7]

— **stitches** in: Tabac.[7]

— **stuffing** at, chronic loud cough from stuffing at epigastrium, chiefly on waking in the morning; has then a fit of coughing and expectoration of tough mucus, with lightness in head: Kali b.[6]

— **tickling** in pit of stomach and larynx causes a dry barking cough; worse at night, also in day when lying down: Nitr. ac.[7]

— — — persistent, gagging cough from tickling in throat, under sternum, or in the stomach: Lach.[7]

— — — — hacking cough: Tarax.[6]

— — — pit of, spasmodic cough: Bar., Bry., Ferr., Nitr. ac.—2.

**STOMACH**, **tickling** in pit of. See p. 102.

— — — — — whooping cough: Ign., Natr. m.—2.

— — — — — — See pp. 143–4.

**STOOLS**, involuntary: Bell.[10]

**STOOPING**, agg. cough: Arg. n.,[7] Hepar.[5]

**STORM**, cough worse before a thunder-storm: Phos.[7]

**STRAINING** cough: Selen.[7]

**STRANGERS**, child coughs at sight of: Phos.[8]

**SUFFOCATION**. See Breathing, pp. 11, 12, and 172.

**SUFFOCATIVE** (choking) cough: Kali iod.[7]

— — Compare with Choking Cough, p. 26.

— — the child becoming quite stiff and blue in the face: *Ipec.*[6]

— — in children with crying: Samb.[7]

— — **larynx**, suffocative, choking cough, at 5 A. M., as if caused by dryness in larynx; was prevented from talking by spasm of chest, accompanied by red face and sweat all over: Kali c.[6]

— — — with swollen larynx: Kali iod.[7]

— — at **night**: Bry.,[16] Cham.,[10] Lyc.,[1] Sil.,[10] Thuja.[6]

— — — cough loose by day, suffocating spells at night: Lyc.[7]

— — **pharynx**, scraping and dryness in pharynx causing a suffocative cough: Cyc.[10]

— — violent, deep-seated, suffocating cough, with expectoration: Guarea.[1]

**SUGAR**, amel. cough: Sulph.[5]

**SWALLOW**, after frequent short, dry cough, must swallow, something seems to rise up in throat; whining and moaning: *Cina.*[14]

**SWALLOWING** excites cough and gagging: Lyssin. (Hg.)

**SWEETS**, cough from eating: Spong.[7]

**TEASING** dry cough, day and night: Kali c.[7]

— dry teasing cough caused by tickling in bronchi, uncovering even a hand: Rhus.[7]

**TEMPERATURE**, a change of agg. cough: Carbo v., Lach.[7]

**THORAX.** See Chest.

**THROAT**, aching in, only during a coughing fit, as if an ulcer would open: Caps.[6]

— **burning** in, excites cough: Bov.

— — —, dry fatiguing cough caused by a violent, almost burning titillation in throat, before retiring in evening: Arg. n.[6]

— — —, on coughing, night: Castor.[6]

— **cracking** in, during cough: Lyc.[1]

— **dryness** in, with cough: Phos.[7]

— **clear**, constant desire to clear the: Crot. t.[1]

— **coldness**, sensation of in: *Brom.*

— **constriction** of, irritation inducing cough, attended by constriction of throat and spasm of chest: Carbo an.[6]

— — —, with cough and amel. by the cough: Asar.[1]

— **contractive** sensation in, cough from, especially at night when sleeping: Nitr. ac.[6]

— **crawling** in, hacking cough from: Caust.[7]

— and tickling in, dry spasmodic cough: Kreos.[6]

— **cutting** in sides of, when coughing: Lyc.

— **darting** sensation in, causes cough: Cistus.[6]

— **dryness** in, excites cough:

**THROAT, dryness**—(*Continued*). Atrop., Eugen., *Kalm.*,[7] Verat. v.—1.

— — itching dryness causes cough, morning: Mang.[6]

— — dry glottis causes cough as from a cold: Hura.

— — of, with cough: Phyt.[6]

— **fauces.** See p. 45.

— **irritation** in, excites hacking cough, much increased by heat and cold air: Hepar.[7]

— — — on inspiration: Asar.,[7] *Rumex.*

— — — on deep or rapid inspiration: *Rumex.* (Dunham.)

— — — night, cough from: Tabac.[1]

— **itching** dryness in, short and hacking cough, morning: Mang.[6]

— **mucus** in, sensation as if tenacious mucus were hanging down in throat, causing hacking cough: Laur.[6]

— **nares**, sensation as of an acrid fluid through posterior nares, excites cough: Kali b.[12]

— — pain in posterior nares, as from air passing through with violence, when coughing or talking: Mag. s.[10]

— **rawness** in, excites cough: Sil.[1]

— — and hoarseness in, disappearing after a few paroxysms of cough: Mag. c.[1]

— **roughness** in, exciting cough: Castor.[6]

— — —, when coughing: Natr. c.[6]

— **scraping** in, excites cough: Mag. m.[6]

— — — afternoon, in open air, cough from: Phos.[6]

— — dry sensation in throat, making it difficult for him to talk and obliging him to cough: Senega.[6]

— — in, morning, cough from: Upa.[1]

**THROAT**, **scraping** in, on coughing: Mag. c.,[1] Petro., Senega.—6.
— **scratching** in, when reading aloud: Nitr. ac.[6]
— **soreness** in, on coughing, after dinner: Phos.
— **stinging** in, with fluent coryza: Lact.[6]
— — and dryness in, with rough cough: Merc.[6]
— — and roughness of, with dry cough: Natr. c.[6]
— **tear**, sensation in throat when coughing, as if it would tear away a piece of flesh: Phos.[6]
— **tickling** in, evening, cough from: Nicc.[6]
— — — 1.30 P. M., cough from: Lac. can.[26]
— — —, gagging, persistent cough from tickling in throat, under sternum, or in stomach; worse on falling asleep or during day, from change of temperature: Lach.[7]
— — —, **morning**, dry cough from tickling in throat, night or day; dry in morning, with stitches in chest or left hypochondrium, becomes loose in afternoon: Amm. m.[7]
— — — — loose cough from: Natr. sul.[6]
— — and scraping in, from below upward, with irritation inducing cough: Tabac.[6]
— — in region of thyroid cartilage, which provokes cough, whereby the tickling is made worse: *Squil.*[13]
— — sensation in upper part of, causing constant hacking cough: Lac. can.[26]
— **titillating** dryness in, cough: Lach.[6]
— — in throat, exciting cough (waking him at night): Phos.[6]

**THROAT**, **titillating**, violent titillation in, obliging him to cough, before dinner, recurring at same hour for several days: Arg. n.[6]
— **touching** (pressure on) excites cough: Bell.,[25] Stram.[5]

**THROAT-PIT**, irritation in, excites cough: Squil.[6]
— **itching** in chest (trachea), and pit of throat with dry cough, which does not alleviate the itching: Phos.[6]
— **roughness** in, with drawing stitches when coughing and sneezing; hawking relieves the symptom: Borax.[6]
— **tickling** in, excites cough: Natr. m.[6]
— — itching in region of pit of throat, threatening suffocation, until a concussive cough sets in, continuing for hours without interruption, and causing pain in abdomen and throat: Sil.[6]
— **tingling** in, cough from, with discharge of tough mucus tasting like grease: Mag. m.[6]

**TONGUE**, hacking cough from putting out: Lyc.[10]

**TRACHEA**, **fullness**, constant feeling of fullness in trachea, with irritation and hacking difficulty of speech, and frequent loose cough: Lact.[6]
— **humming** sound in windpipe, frequent cough with: Natr. c.[6]
— **irritation**, low down in trachea, especially when stooping, cough from: Spig.
— — evening cough, fatiguing chest, occasioned by an irritation in lower part of trachea: Petr.[6]
— **itching** below larynx causing hacking cough: Laur.[6]
— **tough mucus**, in morning tough mucus in lower part of trachea which cannot be dislodged by

**TRACHEA**—(*Continued*).
coughing and hawking; he makes great exertion, but only loosens a little, and even this does not come into mouth, but has to be swallowed; after hawking and coughing a scraping sensation remains in trachea as if it were raw and sore; finally the mucus loosens itself, and he is obliged to hawk it up repeatedly: Cann. s.[1]
— **pressure** on, excites cough: Bell. (Dunham.)
— **prickling**, hacking induced by prickling in larynx and trachea, with subsequent dryness in mouth and larynx: Laur.[6]
— **rawness** and soreness high up in, when coughing: Arg. n.[7]
— **roughness** in, throat and trachea, also with hoarseness, or with irritation which induces hacking cough, or with difficulty of speech, or with drawing in

**TRACHEA**—(*Continued*).
trachea and hacking cough: Laur.[6]
— **tickling** at bifurcation of, exciting cough: Coc. c.[1]
— — in trachea, extending from pit of stomach to epiglottis, cough: Puls.[6]

**WAKED**, hacking cough, wakening at midnight, with scraping in chest and sensation as if stomach would turn, as if vomiting would set in: Ruta.[6]

**WEATHER**, titillating burning or stinging, particularly in open air, during wet and cold weather, with constant irritation in, obliging him to cough, or to hawk, with constant expectoration: Aphis.[6]

**WHEEZING** respiration with cough and frothy expectoration: Ars.
— — See under Respiration.

